# Beyond Conventional: The New Horizon of Anti-Angiogenic microRNAs in Non-Small Cell Lung Cancer Therapy

**DOI:** 10.3390/ijms21218002

**Published:** 2020-10-27

**Authors:** Alexandru Tirpe, Diana Gulei, George Razvan Tirpe, Andreea Nutu, Alexandru Irimie, Paola Campomenosi, Laura Ancuta Pop, Ioana Berindan-Neagoe

**Affiliations:** 1 Faculty of Medicine, Iuliu Hatieganu University of Medicine and Pharmacy, 8 Victor Babes Street, 400012 Cluj-Napoca, Romania; altirpe@gmail.com; 2 Research Center for Functional Genomics, Biomedicine and Translational Medicine, Iuliu Hatieganu University of Medicine and Pharmacy, 23 Marinescu Street, 400337 Cluj-Napoca, Romania; andreeanutu.an@gmail.com; 3 Research Center for Advanced Medicine-Medfuture, Iuliu Hatieganu University of Medicine and Pharmacy, 23 Marinescu Street, 400337 Cluj-Napoca, Romania; diana.c.gulei@gmail.com; 4 County Emergency Hospital Cluj-Napoca, 3-5 Clinicilor Street, 400000 Cluj-Napoca, Romania; razvantirpe@gmail.com; 5 11th Department of Oncological Surgery and Gynecological Oncology, “Iuliu Hatieganu” University of Medicine and Pharmacy, 400015 Cluj-Napoca, Romania; airimie@umfcluj.ro; 6 Department of Surgery, The Oncology Institute “Prof. Dr. Ion Chiricuta”, 34-36 Republicii Street, 400015 Cluj-Napoca, Romania; 7 Department of Biotechnology and Life Sciences, DBSV, University of Insubria, 21100 Varese, Italy; paola.campomenosi@uninsubria.it; 8 Department of Functional Genomics and Experimental Pathology, The Oncology Institute “Prof. Dr. Ion Chiricuta”, 34-36 Republicii Street, 400015 Cluj-Napoca, Romania

**Keywords:** microRNA, miRNA, ncRNA, lung cancer, NSCLC, angiogenesis, cancer therapy

## Abstract

GLOBOCAN 2018 identified lung cancer as the leading oncological pathology in terms of incidence and mortality rates. Angiogenesis is a key adaptive mechanism of numerous malignancies that promotes metastatic spread in view of the dependency of cancer cells on nutrients and oxygen, favoring invasion. Limitation of the angiogenic process could significantly hamper the disease advancement through starvation of the primary tumor and impairment of metastatic spread. This review explores the basic molecular mechanisms of non-small cell lung cancer (NSCLC) angiogenesis, and discusses the influences of the key proangiogenic factors—the vascular endothelial growth factor-A (VEGF-A), basic fibroblast growth factor (FGF2), several matrix metalloproteinases (MMPs—MMP-2, MMP-7, MMP-9) and hypoxia—and the therapeutic implications of microRNAs (miRNAs, miRs) throughout the entire process, while also providing critical reviews of a number of microRNAs, with a focus on miR-126, miR-182, miR-155, miR-21 and let-7b. Finally, current conventional NSCLC anti-angiogenics—bevacizumab, ramucirumab and nintedanib—are briefly summarized through the lens of evidence-based medicine.

## 1. Introduction

Lung cancer remains the most common diagnosed malignancy in the world. In 2018, there were 2.094 million new cases of lung cancer diagnosed worldwide, and 1.761 million deaths caused by this malignancy [1]. Lung cancer is classified according to the histological type. Small cell lung carcinoma (SCLC) accounts for approximately 15% of lung cancers and NSCLC for the remaining 85% [2]. NSCLC is further classified into three histological subtypes: lung adenocarcinoma (AC, LUAD), which accounts for approximately 40%; squamous-cell carcinoma (SCC, LUSC) with 30% of the cases; and large-cell carcinoma (LCC), which represents 10% [3]. Adenocarcinoma is considered to be the most common type of lung cancer, comprising more than 50% of all NSCLC diagnoses [4]. 

NSCLC progression relies on the formation of new blood vessels that provide oxygen and nutrients to cells within the tumor microenvironment (TME), and also enhance the metastatic potential. Angiogenesis is a hallmark of cancer [5] and is largely implicated in the proliferative process and tumorigenesis, and in the physiological growth of blood vessels. Progression of NSCLC to advanced stages is closely linked to the angiogenic process. Angiogenesis is driven by proangiogenic factors such as VEGF, FGF2 and PDGF which bind to specific receptors in order to initiate signaling cascades with a net final effect of new blood vessel formation. Thus, the neoangiogenic process represents an obvious target in cancer therapy by limiting cancer progression. In today’s world, alteration of the neoangiogenic process in oncological patients can be achieved through the inhibition of various pathways by antibodies directed against VEGF or VEGF receptors (VEGFRs) or tyrosine kinase inhibitors (TKIs).

Non-coding RNAs (ncRNAs) such as miRNAs have a great therapeutic potential, as multiple ncRNAs are able to target a number of angiogenesis-related factors. In the current review we discuss a palette of miRNAs that we deem important therapeutic candidates for future advanced therapies, along with the mechanistic implications of these miRNAs. 

## 2. Major Angiogenic Factors Are Regulated by microRNAs

**Figure 1:**
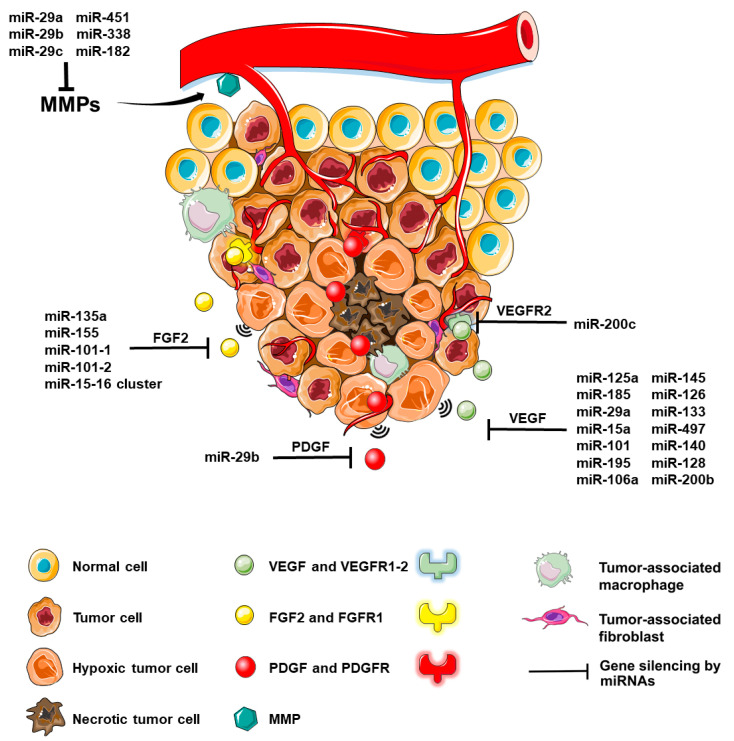
A brief summary of the mechanisms of angiogenesis and modulatory microRNAs. The tumor microenvironment (TME) presented here is composed of tumor cells, hypoxic tumor cells, tumor-associated macrophages, fibroblasts and extracellular matrix. The angiogenic process interests TME-incorporated hypoxic tumor cells situated beyond the Folkman limit, at a distance of 2–3 mm from the nearest arterial blood vessel, which release proangiogenic signaling molecules. The main player, VEGF, is stimulated by these low oxygen levels through the hypoxia inducible factor-1α (HIF-1α) [9], further binding to its receptors, VEGFR1-2. Other key proangiogenic players represented include FGF2 and its receptor, FGFR1 and the PDGF-PDGFR duo. MMPs, along with FGF2, degrade the ECM in order to promote angiogenesis through various mechanisms, including the proteolytic release of angiogenic factors sequestered within the ECM. For integrative purposes, Figure 1 also highlights a selection of miRNAs that target these proangiogenic factors (VEGF, VEGFR2, FGF2, PDGF, MMPs) with silencing effects.

Angiogenesis is a common feature for tumor growth beyond 2 to 3 mm^3^ [6]. The newly formed blood vessels allow the diffusion of nutrients and oxygen to depleted peripheral tumor cells, with concomitant elimination of waste products, thereby facilitating tumor cell survival and tumor growth. These tumor vessels are morphologically and functionally different from normal vasculature, with reduced blood flow, abnormal EC junctions and increased leakiness, which allows tumor cell intravasation [7]. The angiogenic process is regulated at various levels, starting with factors within the TME (e.g., hypoxia [8,9] and extracellular matrix (ECM)), followed by modifications in signaling pathways containing key regulatory molecules (e.g., VEGF expression) and fine-tuning modulation through alterations in the ncRNA profile, including miRNA alterations. MiRNAs are small single-stranded non-coding RNAs with total lengths of 19–25 nucleotides [10] that are able to regulate gene expression through translational repression, cleavage of the mRNA and mRNA decay by rapid-deadenylation [11]. A large number of studies found that translational repression, mRNA deadenylation and the decapping process are the result of the binding of the miRNA to the 3′-UTR region of the target mRNA [12,13]. In the last few decades, numerous studies found that miRNAs are largely implicated in cancers as either tumor suppressors or oncogenic factors, from the onset of the uncontrolled, chaotic growth of the first tumor cells to the metastatic and angiogenic processes, mainly by modulating the pathways implicated in the neoplasm. Recent literature suggests that miRNAs could represent a new approach to cancer therapy [14]. The selective approach generated by miRNA therapy would make them the ultimate therapeutic agents in targeting NSCLC angiogenesis. Figure 1 illustrates an integrative overview of the mechanism of angiogenesis, along with the main proangiogenic factors involved and the corresponding modulatory miRNAs.

**Figure 2:**
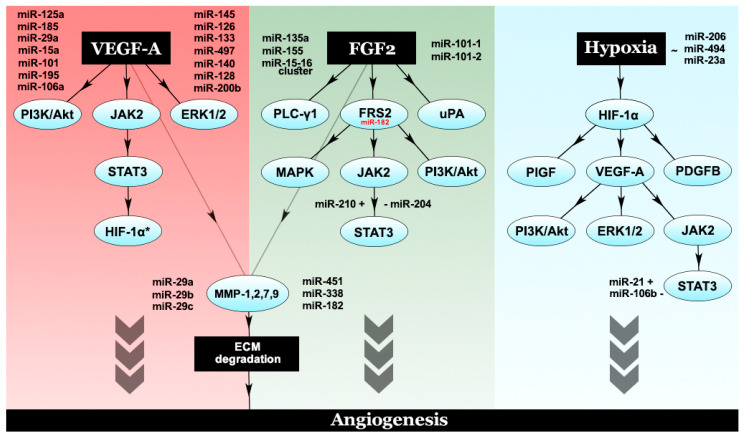
The interplay between different proangiogenic factors and their corresponding modulatory microRNAs. VEGF-A, the chief proangiogenic entity, activates PI3K/Akt, ERK1/2 and JAK2/STAT3. Phosphorylated STAT3 leads to HIF-1α activation, which further increases VEGF-A levels through an autocrine loop. FGF2 activates PLC-γ1, uPA and FRS2. Downstream effectors of FRS2 include the MAPK pathway PI3K/Akt and STAT3. Both VEGF-A and FGF2 are capable of activating a number of MMPs, such as MMP-1,2,7,9, which degrade the ECM and promote angiogenesis. Another major player in angiogenesis is considered to be hypoxia, which exerts its molecular effects through HIF-1α, the hypoxic master regulator. HIF-1α promotes the expression of a number of proangiogenic factors—PlGF, PDGFB and VEGF-A.

Moreover, neoangiogenesis is a complex process driven by the angiogenic switch, an imbalance between proangiogenic factors and anti-angiogenic factors. The most important proangiogenic factors, and suitable therapeutic targets, along with their anti-angiogenic counterparts, are represented in Table 1, along with miRNAs that target these factors, obtained through bioinformatic means. Angiogenesis is driven by the overexpression of these factors rather than the underexpression of the anti-angiogenic factors. The interplay between proangiogenic factors, their corresponding pathways and modulatory miRNAs are illustrated in Figure 2.

### 2.1. VEGF as a Central Player and Its miRNA Regulation

VEGF is the main regulator of the physiological and pathological growth of any type of blood vessel in the human body, acting through vasculogenic and angiogenic processes [29,30]. The VEGF family consists of several members, including the placental growth factor (PLGF); VEGF-A, B, C, D; and the viral VEGF-Es. VEGF-A is the chief proangiogenic factor from the VEGF family, with six isoforms that are able to bind to two tyrosine kinase receptors: VEGFR1 (Flt-1) and VEGFR2 (KDR/Flk1) [31,32]. VEGFR3 regulates lymphangiogenesis [32] and not angiogenesis, whilst VEGFR2 mediates the majority of effects attributed to VEGF through paracrine and autocrine mechanisms, including growth and permeability actions [33].

As such, VEGF production is upregulated in multiple malignancies, including lung cancers [34]. Moreover, the upregulation of VEGF is a marker of poor prognosis in NSCLC [35] and is associated with an enhancement of the angiogenic process. Mechanistically, in NSCLC, VEGF activates several pathways, including PI3K/Akt, extracellular signal-regulated kinase 1/2 (ERK 1/2) and STAT3, which lead to increasing amounts of VEGF through an autocrine loop [36]. The implications of the PI3K/Akt and ERK1/2 pathways have also been highlighted by Ma et al. in a 2019 study, suggesting their vast implications in the modulation of angiogenesis [37]. Moreover, an in vitro study by Xie et al. found that hypoxia is able to induce PI3k/Akt activation in A549 cells; treatment with the PI3K/Akt inhibitor LY294002 had a suppressive effect on VEGF production within the A549 cells in both normoxic and hypoxic conditions, although the inhibition was more potent under normoxic conditions. However, this suppression was not complete, suggesting that other signaling pathways are involved in VEGF expression [38]. Zhao et al. underlined the importance of the JAK2/STAT3 pathway in tumor angiogenesis by upregulating VEGF and FGF2 expression in lung cancer cells. Furthermore, the same Zhao study found a correlation between pSTAT3 expression and VEGF/FGF2 expression, and a JAK2/STAT3 positive association with NSCLC stage and overall survival (OS) [39]. Intrinsically, the vast pathway activation has further stimulatory roles upon neoplastic cells in secreting VEGF [36], creating a regulatory loop. In a study by Shi et al., increased expression of **miR-200c** led to the inhibition of Akt in A549 cell line, through its primary target, the VEGFR2 [40]. Another study by Dodd et al. showed that in the hypoxic TME, phosphorylated STAT3 is able to activate the hypoxia mediator HIF-1α by driving its mRNA transcription [41]. The activated HIF-1α elevates the expression of angiogenic VEGF [9,42]. As such, the aforementioned regulatory loop enhances angiogenesis and induces cancer progression. It is abundantly clear that the intricate mechanisms of angiogenesis can be regulated at various levels by various miRNA entities. 

**MiR-126** acts on multiple target genes involved in lung cancer angiogenesis, such as VEGFA and epidermal growth factor-like domain 7 (EGFL7) [43,44,45]. It is one of the most differentially expressed miRNA in lung cancer [46], with an overall reduced expression. MiR-126 directly targets the 3′-UTR of the VEGF-A gene. Liu et al. compared lung cancer tissue with normal lung tissue and found miR-126 expression to be downregulated in the neoplastic tissue, with increased VEGF-A expression [23]. Moreover, transfection experiments by Liu et al. and Zhu et al. confirmed this finding [23]: miR-126 suppresses VEGF-A expression and inhibits cancer cell growth [45]. Furthermore, Zhu et al. reported the first association between miR-126 expression and cancer chemotherapy; the team found that enhanced miR-126 expression increases the sensitivity of NSCLC cells to therapy through negative regulation of the VEGF-A/PI3K/Akt/multidrug resistance protein 1 (MRP1) pathway. Moreover, the activity of miR-126 on the PI3K-Akt pathway was confirmed in a study by Yang et al. where the team demonstrated that miR-126 overexpression in NSCLC cells reduces cell growth in vitro and also reduces tumor proliferation in a nude mouse xenograft model. The authors identified the 3′-UTR region of the PI3K regulatory beta subunit (PI3KR2) mRNA as a target of miR-126 [47]. A study by Shi et al. concluded that overexpression of miR-126 downregulates VEGF-A, and the expression of the VEGFR2, by inactivating the VEGF-A/VEGFR2/ERK signaling pathway [48], proving its importance in regulating angiogenesis.

Furthermore, studies have shown high expression of **miR-21**, a well-known oncomiR, in the plasma [49] and also in the tissues [50] of NSCLC patients compared to healthy donors. Liu et al. demonstrated that a STAT3 knockdown reduced miR-21 levels in HBE (human bronchial epithelial)-derived exosomes—exosomes are small membrane vesicles secreted by most cell types that are involved in intercellular communication—with consequent inhibition of angiogenesis [51,52]. Exosome-derived miR-21 induces STAT3 activation, increasing VEGF levels in recipient cells and activating angiogenesis. Furthermore, Liu et al. identified a dose-response dependency between miR-21-exosomes and VEGF levels in recipient cells. Inhibition of exosomal miR-21 led to decreased VEGF expression within the recipient cells, proving the modulatory effects of miR-21 in angiogenesis [51]. Recent work by Dong et al. found that enhanced expression of miR-21 might be correlated with the development of brain metastases in patients with NSCLC, as miR-21 had increased expression in the cohort associated with brain metastatic spots. The same authors showed that a knockdown of miR-21 might restrain cell proliferation, migration, invasion and angiogenic properties and might induce tumor cell apoptosis. Moreover, an in vitro experiment found that A549 cells in the miR-21 inhibition group showed almost no tube formation, contrarily to the negative control group and the mock group. Further analysis of tube length difference showed that the inhibition group had statistically significant shorter tube length, and a lower number of junction points compared to the negative control (*p* < 0.05). As such, Dong et al. indicated miR-21’s potential in angiogenic modulation, and its potential as a biomarker for the development of brain metastases in NSCLC [53].

Gu et al. demonstrated the implication of **miR-497** in modulating VEGF-A expression. The authors showed that miR-497 overexpression in lung cancer cell lines reduced VEGF-A expression by binding to the 3′-UTR of VEGF-A mRNA, thereby inhibiting its translation, and subsequently, angiogenesis. Furthermore, their results showed that miR-497 can inhibit NSCLC cell growth and invasion, as miR-497 depletion had the opposite effect, increasing cell invasiveness and growth [54]. 

Another study by Jusufovic et al. reported a significantly decreased expression of **let-7b** and a higher degree of MVD in tumor tissue compared to non-tumor tissue from NSCLC patients. The expression of let-7b has been negatively correlated with MVD and low let-7b expression was significantly correlated with the prognosis, as patients with low let-7b showed shorter progression-free survival (PFS) and OS [22]. **LIN28B**—a RNA-binding protein that regulates let-7 microRNAs—has been shown to be associated with tumor initiation, progression and resistance to therapy in lung cancer. Furthermore, Meder et al. reported that LIN28B increased VEGF-A expression in a KRAS-mutant lung AC [55]. Its expression is associated with a poor prognosis in several solid cancers, including lung cancer, as it enhances the tumor’s angiogenic properties [55,56].

### 2.2. FGF2—A Multifaceted Proangiogenic Factor and Its miRNA Modulation

FGF2 is a member of the fibroblast growth factor family and is frequently found upregulated in different malignancies. Takanami et al. were the first to report the expression of FGF2 in NSCLC [57,58,59]; further studies suggested that increased expression of FGF2 correlates with poor prognosis [57]. Moreover, FGF2 presents a neurotrophic and mitogenic effect [60], whilst also being a potent inducer of the angiogenic process. This mitogenic effect, along with the chemotactic response, requires activation of ERK1/2 and protein kinase C (PKC) pathways [61]. FGF2 plays autocrine roles in ECs, which predominantly express FGFR1, a type of tyrosine kinase receptor that binds the factor. Interactions of these entities induce EC proliferation, migration and angiogenesis. Mechanistically, FGF2 binds FGFR1 with the help of a stabilizing partner such as the heparan sulfate proteoglycan (HSPG) syndecan, leading to signal transmission to two key intracellular substrates—phospholipase C-γ1 (PLC-γ1, FRS1) and FGFR substrate 2 (FRS2). Subsequent phosphorylation of FRS2 activates the Ras-mitogen-activated protein kinase (MAPK), and the PI3K/Akt signaling pathways in cancer cells and ECs, promoting angiogenic processes [61]. Another pathway that is largely involved in FGF2-induced cancer angiogenesis is JAK2/STAT3; a study by Zhao et al. showed that JAK2/STAT3 activation increased FGF2 expression, inducing angiogenesis. Furthermore, treatment of A549 and NCI-H292 cells with AG490, a JAK2 inhibitor, reduced FGF2 expression, proving that the JAK2/STAT3 pathway is associated with FGF2 modulation in lung cancer cells [39]. Additionally, FGF2 induces MMP and urokinase-type plasminogen activator (uPA) synthesis in ECs, further stimulating the angiogenic process by breaking down the extracellular matrix, allowing ECs to migrate [61]. 

Moreover, FGF2 and FGFRs may also play an essential role in anti-angiogenic therapy resistance in cancer. By treating the patient with anti-VEGF agents, the VEGF-dependent vessels will be targeted and disrupted, whilst the tumor vessels will be increasingly covered by pericytes; these pericytes have been shown to overexpress FGF2, thereby switching the angiogenic pathway dependency towards FGF2 [62]. Mechanistically, in late stage tumors, as the resistance to VEGFR2 blockade develops, the malignancy promotes a VEGF-independent angiogenic process that relies on other proangiogenic factors, including FGF2, highlighting the extensive crosstalk between FGF2, VEGF and their receptors [63].

A study by Zhou et al. found that **miR-135a** is able to regulate several proangiogenic factors, including FGF2. The team found that miR-135a levels were lower in NSCLC tissues compared to normal adjacent tissue. Contrarily, IGF-1, PI3K and Akt mRNA levels were higher in the lung cancer tissues. The same authors conducted a series of in vitro experiments on A549 cancer cells that further proved the implication of miR-135a in the regulation of different proangiogenic factors, with an emphasis on FGF2. Accordingly, the authors concluded that miR-135a is able to inhibit angiogenesis by decreasing VEGF, FGF2 and IL-8 levels in an IGF-1-dependent mechanism [60].

**MiR-182**, frequently upregulated in cancers, is mostly viewed as an oncogene, its activity being correlated with the modulation of multiple targets [64], including the anti-angiogenic factor Tsp1. However, contrarily, miR-182 targets FRS2, a downstream member of the FGF pathway [44,65], which is supposedly an inducer of tumor progression in NSCLC through the activation of angiogenesis. Older studies showed that FRS2 plays a role in the transmission of signals from the FGFR to the Ras/MAPK signaling pathway [66]. Moreover, the Ras/MAPK pathway is involved in multiple aberrant cell functions featured in malignancies, including neoplastic angiogenesis. Additionally, activated FRS2 is involved in PI3K/Akt signaling, a well-known modulatory pathway of angiogenesis [61]. We have detailed the interaction of FGF2–FGFR and the involvement of FRS2 in NSCLC angiogenesis in the FGF2-centered mechanistic section above. The overall effect of FRS2-targeting miR-182 is an indirect inhibition of angiogenesis. Stenvold et al. identified a rather weak, but significant correlation between miR-182 and FGF2 [65], further supporting that miR-182 can regulate angiogenesis by its connection with this proangiogenic factor.

Various studies have found **miR-155** to be overexpressed in NSCLC [67,68,69,70]. Two of the studies mentioned beforehand have investigated the prognostic impact of miR-155 in NSCLC, high miR-155 expression being correlated with a poor OS in lung AC and SCC [71,72]. Donnem et al. investigated the correlation between miR-155 and different angiogenic markers in NSCLC, and found miR-155 to be significantly correlated with FGF2 in the studied cohort (r = 0.17, *p* = 0.002). Consequently, miR-155 may be able to modulate angiogenesis through the alteration of FGF2 and its downstream pathways [44]. The mechanism behind FGF2’s action includes the phosphorylation of FRS2 which activates the MAPK and PI3K/Akt signaling pathways in both cancer cells and ECs [61]. Additionally, the JAK2/STAT3 pathway is involved in the FGF2-dependent angiogenic modulation, and MMP secretion [39]. Moreover, Voortman et al. found miR-155 to have no significant prognostic impact in the studied cohort in a qRT-PCR study of formalin-fixed paraffin-embedded NSCLC tumor specimens from 639 resected NSCLC patients who participated in the International Adjuvant Lung Cancer Trial (IALT) [73]. Contrarily, a study by Donnem et al. investigating the prognostic impacts of miR-155 in NSCLC on 335 patients with stage I to IIIA NSCLC found that miR-155 expression had no significant prognostic impact in the total cohort (*p* = 0.43), but depended on the histological subtype. In the same study, high miR-155 expression in ACs had a negative prognostic effect on survival in univariate analysis (*p* = 0.086) and was found to be an independent prognostic factor in multivariate analysis (HR 1.87, CI 95% 1.01–3.48, *p* = 0.047). Moreover, in SCC patients with lymph node metastasis, miR-155 was found to have a positive prognostic impact on survival in both univariate analysis (*p* = 0.034) and multivariate analysis (HR 0.45, CI 95% 0.21–0.96, *p* = 0.039) [72].

Another study by Fan et al. identified **miR-210** as a proangiogenic microRNA. In their experiment, Fan et al. showed that exosomes containing miR-210 stimulated angiogenesis through a cancer-associated fibroblasts (CAF)-dependent mechanism. In addition, an in-depth analysis found that miR-210 was able to increase the expression of several proangiogenic factors, including FGF2, VEGF-A and MMP9, by activating a number of pathways involved in the angiogenic switch, such as JAK2/STAT3 and ten-eleven translocation 2 (TET2). Concomitantly, the same study found that miR-210 was overexpressed in serum exosomes of patients with untreated NSCLC, further proving the implication of this microRNA in lung cancer angiogenesis [74].

### 2.3. The Extracellular Matrix: Support for Angiogenesis

The TME is an important consideration in the molecular biology of cancer. It comprises, among others, cancer cells, a large palette of non-cancer cells, blood vessels and ECM entities [75]. Degradation of the ECM entities which support the TME allows ECs to migrate, positively influencing the angiogenic process. In lung cancer, the hypoxic TME enhances the transcription of a number of proangiogenic factors, growth factors and MMPs [76]. Furthermore, the activation of MMPs can be induced by a number of angiogenic factors—VEGF, FGF2, TGF-α,β and angiogenin [77]. These MMPs poses angiogenic activity by degrading the surrounding ECM.

An old study by Itoh et al. found that mice who are MMP-2 deficient show reduced tumor angiogenic properties [78]. Moreover, an in vitro study by Chetty et al. showed that there was a connection between MMP-2 and lung cancer cell tube formation of ECs—MMP-2 siRNA inhibited tube formation—whilst recombinant human-MMP-2 induced angiogenic properties [79]. Mechanistically, MMP-2 inhibition decreased HIF-1α levels and disrupted PI3K-dependent VEGF expression. The authors concluded that the transcriptional suppression of MMP-2 may be able to decrease neoangiogenesis by downregulating VEGF-A [79]. Another study by Chetty et al. found that MMP-2 inhibition in lung tumor cells leads to an inhibition of VEGF-related activation of AKT in ECs, followed by increased ERK activation and an inductive effect on TIMP-3 [80]. TIMPs (TIMP1-4) play an essential role in the regulation of ECM by their inhibitory action on MMPs. Specifically, TIMPs hamper neoangiogenesis by inhibiting MMPs [77] with a consecutive sequestration of ECs in the ECM, further proving the intricate mechanisms of ECM in the regulation of angiogenesis.

In addition, a study by Blanco-Prieto et al. researched serum MMPs (MMP-1, MMP-2, MMP-7, MMP-9, MMP-10) and TIMP-1 levels by multiplexed immunoassays in a study cohort of 19 NSCLC cases and 19 healthy controls. The authors found that MMP-1, MMP-7 and MMP-9 serum levels were slightly elevated in NSCLC patients, prompting the team to rely on larger cohorts for the evaluation of these MMPs as biomarkers for NSCLC. MMP-2 and MMP-10 were discarded from the study because of the low AUC levels, showing poor diagnostic capacity, in spite of their importance in angiogenesis. Blanco-Prieto et al. found that although MMP-1 serum levels were higher than control, the discrimination was poor, highlighted by an AUC value of 0.538; further reference studies suggest that MMP-1 may be implicated in the late stages of the neoplastic disease [81,82]. The team also studied MMP-7 levels in a larger population, showing that MMP-7 serum levels were significantly increased in NSCLC patients with a moderate discrimination level (AUC value of 0.604). Last but not least, MMP-9 serum levels were found to be significantly elevated in NSCLC patients compared to both healthy controls and patients with benign pulmonary pathologies. Blanco-Prieto et al. concluded that MMP-9 serum levels offered the best diagnostic capacity for NSCLC (AUC value of 0.739) of the studied entities [81]. Other studies support the importance of high MMP-9 levels in NSCLC [83,84].

Moreover, Stenvold et al. identified a correlation between **miR-182** and MMP-7 in a NSCLC cohort. The examination of coexpression of the miR-182 and MMP-7 resulted in patients with high miR-182 and high MMP-7 expression having independently better survival compared to those with low miR-182 and low MMP-7 expression (hazard ratio (HR) = 0.49, *p* = 0.015). SCC patients with high/high expression levels have far better prognoses compared to those in the rest of the groups (HR = 0.26, *p* = 0.012) [65].

### 2.4. Hypoxia Modulates the Angiogenic Process in Lung Cancer

As mentioned beforehand, hypoxia is a key factor in the modulation of angiogenesis [8,9]. Hypoxia induces vast effects in the TME due to the increased oxygen consumption and requirement caused by the proliferative process.

The majority of hypoxia-derived effects are orchestrated by HIF-1, the hypoxic master regulator; HIF-1, through HIF-1α and HIF-2α, binds to the hypoxia responsive element (HRE) located in the promoter region of the chief proangiogenic factor, VEGF [9], activating the downstream PI3K/Akt, ERK1/2 and JAK2/STAT3 pathways and promoting angiogenesis [37,39]. We have previously shown that the autocrine loop created further stimulates VEGF production. Low oxygen levels can also stimulate a number of other proangiogenic entities, such as FGF and HGF, which promote EC proliferation and enhance migration [85], Angpt2 in a HIF-2α-dependent manner, PLGF, PDGF-β and others. Several studies showed that HIF-1α and HIF-2α have completing effects in the angiogenic setting—the first drives blood vessel growth while the latter induces maturation [86,87]. Tumor hypoxia combined with high serum levels of angiogenesis markers have previously been associated with poor prognosis in NSCLC [88]. The mechanistic interrelation between angiogenesis and hypoxia has been extensively characterized in a previous paper by our group [9].

In a study by Xue et al., **miR-206** was able to decrease the angiogenic ability in NSCLC by inhibiting a specific pathway: 14-3-3ζ/STAT3/HIF-1α/VEGF. Precisely, the team showed that miR-206 targets 14-3-3ζ and thus inhibits the downstream STAT3/HIF-1α/VEGF pathway, impeding the angiogenic process [42]. Furthermore, another study conducted by Mao et al. identified miR-494 as an angiogenic promoter in human vascular endothelial cells (HUVECs), and in the A549 NSCLC cell line. **MiR-494** effectively targets PTEN and thus the consecutive activation of Akt/eNOS pathway. The same team found that hypoxia induces miR-494 expression, perhaps via a HIF-1α-dependent mechanism [89].

Moreover, a study by Hsu et al. exemplified the intricate mechanisms behind hypoxia-dependent miRNA regulation of angiogenesis. In the Hsu study, the hypoxic lung cancer cell-derived exosomes containing **miR-23a** reduced PHD2 expression by directly targeting the 3′-UTR of PHD2 in HUVECs, leading to enhanced HIF-1α activity. Concomitantly, miR-23a increased HIF-1α transcription under both hypoxic and normoxic conditions, with a subsequent activation of angiogenesis. The Hsu study found that the hypoxic lung cancer cell-derived exosomes containing miR-23a disrupted the endothelial barrier by targeting ZO-1 and increased the number of tumor vessels, further proving its implication in lung cancer angiogenesis [90].

### 2.5. miRNAs with Regulatory Potential in Lung Cancer

**Figure 3:**
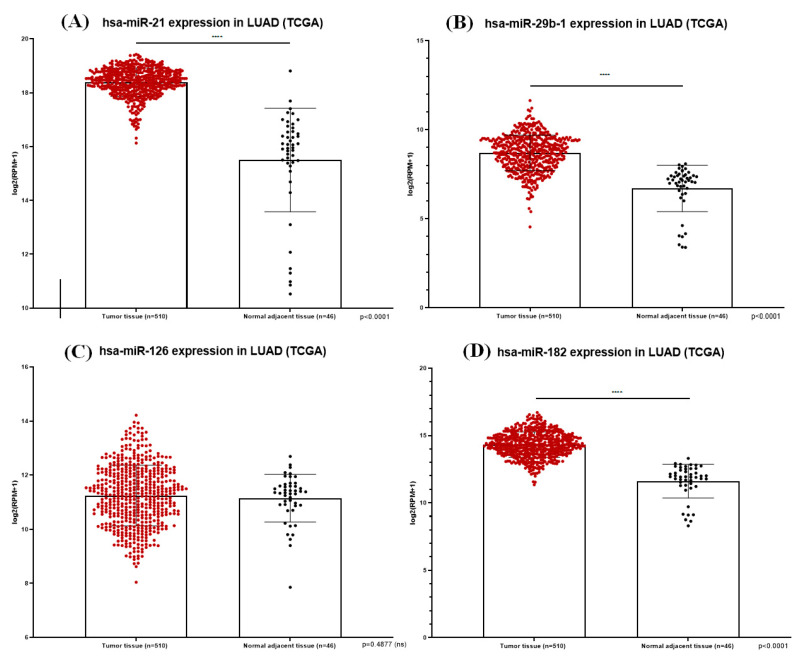
MiR-21 (**A**), miR-29b-1 (**B**), miR-126 (**C**) and miR-182 (**D**) expression in LUAD tissue samples from TCGA database; the plots show statistically significant overexpression of miR-21, miR-29b-1 and miR-182 with *p* < 0.0001

Table 2 presents a short description of a selection of miRNAs that drive angiogenesis in lung cancer, with information varying from miRNA expression and the target gene, to the study type and overall action: miR-106a, miR-141, miR-378, miR-15-16 cluster, miR-106b, miR-128, miR-200b, miR-497 and miR-29b. For integrative and coordination purposes, Table 2 also summarizes the palette of miRNAs discussed in the sections above. Figure 3 illustrates the expression of miR-21, miR-29b-1, miR-126 and miR-182 in LUAD tissue samples from TCGA database, with an obvious dysregulation.

## 3. Current and Future Anti-Angiogenic Therapeutic Options in NSCLC

Several anti-angiogenic drugs have been approved for use in NSCLC, such as monoclonal antibodies directed against VEGF-A/VEGFRs and small molecule tyrosine kinase inhibitors (TKIs). New prospects include the use of ncRNAs that interfere either directly with factors implicated in angiogenesis, or indirectly with intermediary factors in various pathways linked to the angiogenic process. Our exhaustive search from within the literature has not identified any angiogenesis-related miRNAs in use or in clinical trials, and no combinations of standard anti-angiogenic therapy with miRNA-based therapy.

### 3.1. Conventional Anti-Angiogenics

A summary of the conventional anti-angiogenics used in the treatment of NSCLC is presented within Table 3.

### 3.2. MiRNAs as Therapeutic Agents: Prospects

In the past few years, the potential of miRNAs as therapeutic agents increased significantly, which is reflected in the number of publications. However, there are no clinical trials that use miRNAs to target angiogenesis in non-small cell lung cancer at the time of submitting this manuscript. To our knowledge, only two phase I clinical trials evaluated the safety of miRNA-based drugs that included NSCLC patients—the MRX34 clinical trial (clinicaltrials.gov identifier: NCT01829971) and the MesomiR 1 trial (clinicaltrials.gov identifier: NCT02369198). These trials represent a starting point for future research regarding the therapeutic and translational potential of these ncRNAs. Recent results from MRX34 and MesomiR 1 suggest that miRNA-based therapy is effective in a selected pool of patients, taking into consideration the inflammatory adverse effects that may lead to severe complications, as exemplified in the MRX34 study [114].

Considering the aforementioned, we find that the modulation of miR-126 and miR-21 presents great potential as a therapeutic strategy. A valuable argument is that both these miRNAs target multiple pathways involved in NSCLC progression and angiogenesis, with a suggestive increased overall effect.

## 4. Conclusions

The silent progression of NSCLC is one of the crucial factors that shape this malignancy. Folkman’s hypothesis led to the development of a new therapeutic idea in cancers, creating a new game plan in the treatment of NSCLC. To this day, inhibition of angiogenesis represents a viable option in the therapeutic approach of NSCLC.

The identification of the main factors that drive the angiogenic process, along with the corresponding pathways, allowed researchers to build new therapeutic strategies. Essentially, current anti-angiogenic therapy targets either key proangiogenic factors such as VEGF and VEGFR1-2, or intermediaries in the signal transduction cascade. Key proangiogenic factors include VEGF, FGF2, PDGF and others. Current approved anti-angiogenic therapies used in NSCLC consist of either monoclonal antibodies directed against VEGF-A (bevacizumab) and VEGFR2 (ramucirumab), or small-molecule tyrosine kinase inhibitors that bind proangiogenic factors’ receptors, such as the EMA-approved nintedanib which has a multi-targeted profile. To date, small-molecule TKIs have failed to demonstrate any significant improvement in overall survival, with the exception of the EMA-approved nintedanib. A number of studies proved that the efficacy of some anti-angiogenics plus standard chemotherapy is greater than chemotherapy only, rendering their importance in treating NSCLC.

A rather new but not fully explored path in anti-angiogenic therapy is represented by ncRNAs. The paramount role of the miRNAs in the angiogenic process would make them the ultimate therapeutic agents. In this review, we have critically summarized the specific miRNA palette that regulate the angiogenic process in NSCLC and that may present a therapeutic starting point for future research related to anti-angiogenic therapeutics. For instance, miR-126 is able to downregulate VEGF-A levels and thus suppress the angiogenic process [23]; on the other hand, miR-21 is an oncomiR that can be targeted. We suggest that miR-126 and miR-21 present a great therapeutic potential in NSCLC angiogenesis. At the time of submitting this manuscript, there are no clinical trials that use miRNAs in order to target the angiogenic process in NSCLC. The MRX34 and MesomiR 1 clinical trials proved that miRNA therapeutics may benefit carefully selected patients; these clinical trials represent the starting point for future research in this domain. As such, the therapeutic potential of miRNAs in the inhibition of angiogenesis is of great importance and should be further exploited in order to improve patient survival.

## Figures and Tables

**Table 1:** The main proangiogenic and anti-angiogenic factors, along with a selection of miRNAs that target their genes. In general terms, miRNAs compiled in this table which target the proangiogenic factors are downregulated in the context of angiogenic non-small cell lung cancer (NSCLC), whilst miRNAs that target anti-angiogenic factors are upregulated. The factors were compiled after [15,16,17]. The gene mutation frequency was obtained through TCGA analysis. miRNAs were selected through a clear methodology: (1) The factors were searched in the miRTargetLink Human tool (Saarland University) which returned a number of miRNAs; only miRNAs with strong evidence were taken into consideration; (2) these miRNAs were inserted into the OncoMir Cancer Database from the Masonic Cancer Center, University of Minnesota which uses TCGA-based data; (3) the tissue type that was taken into consideration consisted of LUAD and LUSC; (4) the resulting heatmaps were used to evaluate the implications of the aforementioned miRNAs.

Gene	Gene Mutation Frequency ^1^	miRNAs Regulating the Gene ^2^	References ^5^
Proangiogenic			
*VEGFA*	<0.1%	miR-125a, miR-185, miR-29a, miR-101, miR-15a, miR-195, miR-140, miR-145, miR-126, miR-133	[18,19,20,21,22,23]
*FGF2*	No mutations identified	No strong evidence miRNAs identified in the database searchmiR-101-1 ^3^, miR-101-2 ^3^	NR
*PDGFB*	0.3%	miR-29b ^4^	NR
*EGF*	No mutations identified	No strong evidence miRNAs identified in the database searchmiR-3188 ^3^	NR
*TGFB1*		miR-144, miR-29b ^4^	NR
*IL8*		miR-520b	NR
*MMP2*		miR-451, miR-29a, miR-29b ^4^, miR-29c, miR-338, miR-451	[24,25,26]
Anti-angiogenic			
*THBS1*	No mutations identified	miR-182	NR
*TIMP1*		miR-1293	[27]
*IL1A*		No strong evidence miRNAs identified in the database searchmiR-181b-2 ^3^	[28]
*COL18A1*		No strong evidence miRNAs identified in the database searchmiR-450a-1 ^3^, miR-450a-2 ^3^, miR-1292 ^3^, miR-877 ^3^	NR

^1^ TCGA analysis on 1144 NSCLC samples; ^2^ marked as strong evidence on the miRTargetLink Human tool (Saarland University). ^3^ Weak support, warrants further study. ^4^ miR-29b levels can be either increased or decreased. ^5^ miRTargetLink Human–OncoMir analysis along with literature references. NR = no relevant references identified in the literature, but miRNAs identified in the analysis.

**Table 2:** miRNAs with regulatory potential in lung cancer.

miRNA	Expression	Study Type	Target	Overall Action	References
miR-106a	▲	[44]—*ex vivo* (tissue) [67]—*ex vivo* (tissue) [91]—*in vitro* [92]—clinical/*ex vivo*	Predicted: VEGF, FGFR2, STAT3	Upregulated during hypoxia in breast and colon cancer. Augmented expression in NSCLC.	[44,67,91,92]
miR-141	▲ in [93]	[93]—*in vitro*, *ex vivo* (tissue) [94]—*in vitro*, *in vivo*, *ex vivo* (tissue)	KLF6, NRP1, GAB1, CXCL12β, TGFβ2, GATA6	In a lung adenocarcinoma model, overexpression of miR-141 inhibited KLF6 and consecutively increased VEGF-A levels, thus promoting angiogenesis. Opposite results were obtained by Dong et al. who found that miR-141 overexpression exhibits anti-angiogenic properties by suppressing endothelial cell proliferation, migration and tube formation. These effects are the result of miR-141 acting on NRP1, GAB1, CXCL12β, TGFβ2 and GATA6. However, Dong et al. proposed that the effects may vary in different tumor environments.	[93,94]
miR-155	▲	[44]—*ex vivo* (tissue) [67]—*ex vivo* (tissue)	Predicted: FGF2	MiR-155 has been significantly correlated with FGF2 and with a poor OS in lung AC and SCC.	[44,67,71]
miR-182	▲	[44]—*ex vivo* (tissue) [64]—*in vitro*, *ex vivo* (tissue) [65]—*ex vivo* (tissue)	FRS2, Tsp1	The action of miR-182 is intricated, acting on at least 2 targets with antagonizing effect. However, miR-182 is mostly viewed as an oncogene by suppressing the antiangiogenic Tsp1 and thus promoting angiogenesis.	[44,64,65]
miR-21	▲	[51]—*in vitro*, *ex vivo* (serum) [53]—*in vitro*, *ex vivo* (blood)	HIF-1α, PTEN, PDCD4, hMSH2	MiR-21 is a well-known oncomiR with various implications in lung cancer. A study by Liu et al. showed that exosome-derived miR-21 induces STAT3 activation, increasing VEGF levels and thus activating angiogenesis. Furthermore, the X study showed that A549 cells within the miR-21 inhibition group showed almost no tube formation when compared to the control group and the mock group.	[51,53,95]
miR-210	▲	[74]—*in vitro*, *in vivo* [96]—*in vitro*	Succinate dehydrogenase complex subunit D (SDHD), E2F3	MiR-210 exhibits its proangiogenic effects through a CAF-associated mechanism. A study by Fan et al. showed that miR-210 was able to increase the expression of FGF2, VEGFA and MMP9 by activating JAK2/STAT3 and TET2 pathways.	[74,96]
miR-221/222 cluster	▲	[97]—*in vitro*, *in vivo* [98]—*in vitro*, *in vivo*, *ex vivo* (tissue)	TIMP-3 PTEN	MiR-221/222 are over-expressed in NSCLC cells. The cluster suppresses PTEN and TIMP-3 expression, inducing migration and invasiveness. Janssen et al. proved that TIMP-3 knockdown tumors had a higher level of vascularization versus control.	[97,98]
miR-23a	▲	[90]—*in vitro*, *in vivo*, *ex vivo* (tissue)	PHD2, ZO-1	The Hsu study found that miR-23a increases tumor angiogenesis in both hypoxic and normoxic environment. MiR-23a is able to directly target the 3′-UTR of PHD2 in HUVECs, leading to enhanced HIF-1α activity with proangiogenic features. Furthermore, Hsu et al. showed that miR-23a can target ZO-1, disrupting the endothelial barrier and promoting angiogenesis.	[90]
miR-378	▲	[99]—*in vitro*, *in vivo*, *ex vivo* (tissue) [100]—*in vitro*, *in vivo*, *ex vivo* (tissue) [62]—*in vitro*, *in vivo*	RBX1, CRKL	MiR-378 is upregulated in highly invasive lung cancer sub-cell lines. MiR-378 promotes invasion, metastasis through EMT (RBX1, CRKL) and angiogenesis *in vivo*. A study by Ho et al. showed that RBX1 intervenes in HIF-1α pathway to produce VEGF with a subsequent inductive effect on angiogenesis. Accumulating evidence suggests that miR-378 could act as both oncogene and tumor suppressor.	[62,99,100]
miR-494	▲	[89]—*in vitro*, *in vivo*	PTEN	MiR-494 promotes angiogenesis in HUVECs/A549 cells and effectively targets PTEN with the consequent inhibition of the Akt/eNOS pathway.	[89]
miR-15-16 cluster	▼	[101]—*in vitro*, *in vivo*, *ex vivo* (tissue)	FGF2	In a study conducted by Xue et al., hypoxia repressed the miR-15-16 cluster, with a loss of restriction of its target gene, FGF2. This action promoted tumor angiogenesis and metastasis.	[101]
miR-106b	▼	[102]—*in vitro*, *in vivo* [103]—*in vitro*	STAT3	MiR-106b exhibits anti-angiogenic effects by inhibiting STAT3 in ECs. Niu et al. found that VEGF expression correlated positively with STAT3 activity in different human cancer cell lines.	[102,103]
miR-128	▼	[104]—*in vitro*, *in vivo*, *ex vivo* (tissue)	VEGF-A, VEGF-C, VEGFR-2, VEGFR-3	MiR-128 was significantly downregulated in NSCLC tissues and cancer cells and was correlated with NSCLC differentiation, stage and metastasis to lymph nodes. Overexpression of miR-128 in NSCLC cells decreased expression of VEGF-A, VEGFR-2, VEGFR-3, in *in vitro* and *in vivo* experiments.	[104]
miR-200b	▼	[105]—*in vitro*	VEGFA, FLT/VEGFR1, KDR/VEGFR2, Ets1	MiR-200b binds to the 3′-UTR of Ets-1 mRNA to induce translational repression. Ets-1 is a key transcription factor known for its role in promoting angiogenesis. Physiological levels of miR-200b have an inhibitory effect on angiogenesis. In hypoxic conditions, miR-200b downregulation cancels Ets-1 repression, thus promoting angiogenesis. MiR-200b also targets VEGF and its receptors.	[105]
miR-206	▼	[42]—*in vitro*, *in vivo*, *ex vivo* (tissue) [106]—*in vitro*, *ex vivo* (tissue)	SOX9, 14-3-3 ζ	A study by Zhang et al. found downregulated levels of miR-206 and concluded that miR-206 may act as a tumor suppressor partly by targeting SOX9. In another study by Xue et al., miR-206 decreased the angiogenic ability in NSCLC by inhibiting the 14-3-3 ζ/STAT3/HIF-1α/VEGF pathway.	[42,106]
miR-497	▼	[107]—*in vitro*, *in vivo*, *ex vivo* (tissue)	HDGF, FGF2	MiR-497 is downregulated in NSCLC tumors and cell lines. Ectopic expression inhibited cell proliferation and angiogenesis in a SCID mouse xenograft model.	[107,108]
miR-126	▼	[23]—*in vitro*, *in vivo* [43]—*in vitro*, *in vivo* [46]—*in vitro*, *ex vivo* (tissue) [47]—*in vitro*, *in vivo*, *ex vivo* (tissue) [48]—*in vitro*, *ex vivo* (tissue)	VEGFA, EGFL7, PI3KR2	MiR-126 is one of the most differentially expressed miRNA in lung cancer, with an overall reduced expression. A number of studies found that miR-126 targets the VEGFA with a silencing effect. Enhanced miR-126 expression increases the sensitivity of NSCLC cells to chemotherapy through the VEGFA/PI3K/Akt/MRP1 pathway. Furthermore, two studies showed that miR-126 may target PI3KR2 and that by targeting VEGFA it inactivates the VEGFA/VEGFR2/ERK signaling pathway.	[23,43,46,47,48]
miR-135a	▼	[60]—*in vitro*, *ex vivo* (tissue)	IGF-1	The Zhou study identified IGF-1 as a direct target of miR-135a. Zhou et al. showed that miR-135a decreased the angiogenic factors VEGF, FGF2 and IL-8 in the A549 cell line by IGF-1 inhibition.	[60]
miR-29b	▼/▲ (TCGA analysis)	[24]—*in vitro*, *ex vivo* (tissue)	MMP-2, PTEN, PDGFB, TGF-β1	MMP-2 is a known promoter of angiogenesis. In a paper by Wang et al., bioinformatics analysis combined with a polymerase chain reaction study suggested that MMP-2 and PTEN may represent important targets of miR-29b. The same authors concluded that miR-29b behaves as a tumor metastasis suppressor through MMP-2 inhibition. Our analysis found that PDGFB and TGF-β1 may also be targets of miR-29b, marking it as a therapeutic candidate.	[24]
miR-204	▼ (TCGA analysis)	[109]—*in vitro*, *in vivo*	Predicted: JAK2/STAT3	MiR-204 functions as a tumor suppressor in LUAD; in the Liu study, miR-204 promoted cancer cell apoptosis and inhibited cell migration and proliferation *in vitro*, and tumor growth in the *in vivo* model. Conditioned media from A549 cancer cell line with overexpression of miR-204 hampered tube formation and migration of HUVECs. The same authors found decreased levels of HIF-1α, VEGF, PDGF in the A549 cells transfected with miR-204 mimics. Liu et al. concluded that miR-204 inhibits angiogenesis in LUAD, probably via the JAK2/STAT3 pathway.	[109]

▲—upregulated/▼—downregulated.

**Table 3:** Therapeutic agents that target the angiogenic process in lung cancer. Status by the FDA refers to approval in NSCLC.

Therapeutic Agent	Type	Target	Mechanism of Action	Status by the FDA	References
Bevacizumab	Monoclonal antibody	VEGF-A	Recombinant humanized monoclonal antibody directed against VEGF-A.	Approved in combination with carboplatin and paclitaxel chemotherapy for first-line treatment of unresectable, locally advanced, recurrent or metastatic non-squamous NSCLC.	[110]
Ramucirumab	Monoclonal antibody	VEGFR2	Fully humanized monoclonal antibody that specifically binds to VEGFR2, inhibiting angiogenesis.	Approved in combination with docetaxel for metastatic NSCLC with progression after platinum-based chemotherapy. Approved by FDA in May 2020 in combination with erlotinib for first-line treatment of metastatic NSCLC with EGFR exon 19 deletions or exon 21 (L858R) mutations in accordance to the RELAY trial (ClinicalTrials.gov identifier: NCT02411448).	[111]
Nintedanib	Small-molecule, multi-targeted TKI	VEGFR1-3; FGFR1-3; PDGFR-α, β; Src family	Nintedanib inhibits downstream signaling by binding to the adenosine triphosphate (ATP) sites of proangiogenic receptors.	Approved in combination with docetaxel for the treatment of locally advanced, recurrent or metastatic lung adenocarcinoma ^1^.	[112,113]

^1^ regulated by EMA and not by FDA.
